# Efficacy of probiotic use in acute rotavirus diarrhea in children: A systematic review and meta-analysis

**Published:** 2015

**Authors:** Elaheh Ahmadi, Reza Alizadeh-Navaei, Mohammad Sadegh Rezai

**Affiliations:** 1 Young Researchers and Elite Club, Science and Research Branch, Islamic Azad University, Tehran, Iran.; 2 Molecular and Cell Biology Research Center, Mazandaran University of Medical Sciences, Sari, Iran.; 3 Pediatric Infectious Diseases Research Center, Mazandaran University of Medical Sciences, Sari, Iran.

**Keywords:** Probiotics, Rotavirus, Acute Diarrhea, Children

## Abstract

**Background:**

Probiotic therapies with different strains demonstrated some beneficial effects, although some studies did not show any significant effects. This study assessed systematically the current knowledge on the effect of probiotic bacteria on duration of acute rotavirus diarrhea in children compared with control.

**Methods:**

The PubMed, Cochrane Controlled Trial Register (CCTR) and Ovid (Wolters Kluwer Health) were searched between 1980 to June 15, 2013. Randomized controlled trials including the administration of probiotics for treatment of rotavirus diarrhea in infants and children were reviewed.

**Results:**

A total number of 1244 articles were found through the aforementioned search. 203 articles were selected after the first screening of title and abstract. The intervention group included subjects who received probiotic strains and dosage in any conditions. Placebo or any similar vehicle without probiotic was used in the controlled trials. Finally, 14 articles were selected. The outcomes from each study were considered in the duration of diarrhea. Statistical analyses were performed with Stata software. The pooled estimate of efficacy of probiotics in prevention or treatment of disease yielded in all studies a mean difference of 0.41 (CI 95%: -0.56 to –0.25; p<0.001). The pooled estimate of efficacy of l*actobacillus rhamnosus* GG and other probiotics significantly reduced the duration of diarrhea. Among trials, the overall reduction of LGG was 0.47 (CI 95%: -0.80 to -0.14; P= 0.020).

**Conclusion:**

In conclusion, probiotics exert positive effect in reducing the duration of acute rotavirus diarrhea compared with control.

Diarrhea is one of the major causes of morbidity and mortality in both developed and developing countries (1, 2). Rotavirus has been recognized as the most common cause of severe diarrhea in children and infants all over the world since the 1970s (3, 4). Annually, 600,000 child deaths from rotavirus occurred in children under 5 years globally (5, 6). Nearly 85% of rotavirus-associated-diarrhea are observed in the poorest regions of Africa and Asia (7-12). The treatment of rotavirus diarrhea has remained approximately unchanged over the past 35 years (4). Oral rehydration, breast feeding, and early refeeding are still the most important approaches in the control of rotavirus diarrhea in infants and children (3). Several vaccines are currently being used against rotavirus infection; although challenges to vaccination still remains to be resolved (13, 14). Adjuvant therapy has been examined for oral rehydration solution (ORS) with probiotics since 1998 (1, 15).

Recently, probiotic therapy has been investigated in several studies in which the therapeutic effect on rotavirus-associated diarrhea in children was distinguished (16-18), so they have been included in the recent guidelines for the management of acute diarrhea in children of the European Society for Pediatric Infectious Diseases (ESPID) (19-22). Probiotics are defined as live microorganisms which when administered in adequate numbers confer a health benefit on the host (23-26). A previous study concluded that pooled estimates found that probiotics offer a safe and effective method to prevent and treat acute pediatric diarrhea (27). The mechanisms responsible for the beneficial role of probiotics, are studies that have documented direct antimicrobial effects, improve mucosal barrier function, and immunomodulating activities due to the effects of probiotics on both innate and adaptive immunity (28, 29). *Lactobacillus*, b*ifidobacterium* and s*accharomyces *are the most commonly used probiotic strains in the treatment of diarrhea, but other microorganisms, including e*nterococcus*, s*treptococcus*, *Escherichia coli* species have also been used (21, 30). 

After oral administration, probiotic bacteria remain transiently in the human intestine. The efficiency of probiotic, bacteria in the treatment of infectious diarrhea in adults and infants was shown in several studies (31-33). In some studies, the efficiency of probiotics in reducing the course of acute diarrhea in young children was attributed to the consumption of fermented milk (24, 34-36). In some research studies, lactobacillus GG was effective in the treatment of rotavirus diarrhea (26, 31, 35, 37-40). Whereas, lactobacillus acidophilus and bifidobacteria did not manage rotavirus diarrhea in some studies (1). Probiotic therapies with different strains of bacteria indicated some beneficial effects, although some studies did not show any significant effects (20). In this regard, the aim of this study was to review systematically the current knowledge on the effect of probiotic bacteria on duration of acute rotavirus diarrhea in children compared with control.

## Methods

**Figure 1:**
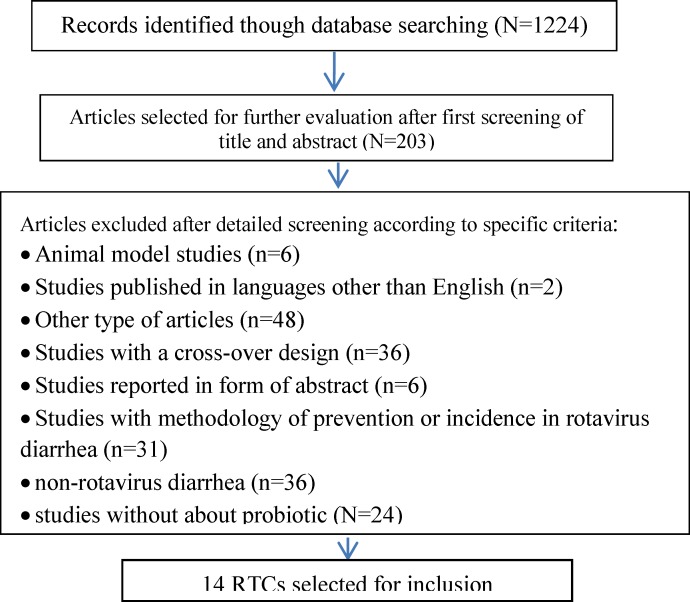
Flow design of the identification eligibility trial for inclusion

The papers in PubMed, Cochrane Controlled Trial Register (CCTR) and Ovid (Wolters Kluwer Health) which were published between 1980 to June 15, 2013 were searched. Furthermore, the references of other clinical trial and review articles have been searched. The search terms included “probiotic”, “treatment”, “rotavirus” and “diarrhea”. A total number of 1244 articles were generated through the aforementioned search. 203 articles were selected after the first screening of title and abstract. The graphical demonstration of the process of opting eligible trails is presented in figure1.

Randomized controlled trials (RTCs) that administer probiotics for treatment of rotavirus diarrhea in infants and children were included in this review. The intervention group was subjected to receive probiotic strains and dosage in any conditions. Placebo or any similar vehicle without probiotic was used in the controlled trials. Moreover, abstract studies and non-randomized controlled trial (non-RCT) articles as well as studies published in languages other than English were excluded from the review. In addition, the present review did not deal with the studies carried out through methodology of prevention or incidence of rotavirus diarrhea, non-rotavirus diarrhea, and antibiotic-associated diarrhea, animal model studies. Consequently, 14 articles were selected regarding these exclusion criteria. 

For their reviews, the outcomes were abstracted data from each study using outcomes that included duration of diarrhea. The length of time diarrhea lasts often depends on what caused it. We surveyed the duration agent in these trials, since the results of frequency are insufficient.

The full articles extracted from the selected studies including the inclusion criteria were reviewed by two persons (M.S.R) and (E.A), and the reviewers assessed the data extraction independently and entered the data into a computer program. All studies were examined according to the list: author, year of publication, study design, age of patients, type of intervention (strain, dose, duration and vehicle), control group, concomitant treatment, diarrhea duration and the outcomes described above them that showed in tables 1.

To measure the duration of diarrhea, each study was analyzed separately. Trials were divided into three main subdivisions. Measurements of diarrhea duration were converted to days, maintaining the number of significant digits in the original units of time. We could not calculate frequency, since the frequency symptom was not reported in major trials. We calculated an absolute difference between probiotics and control groups for each of the outcomes in each study. In the meta-analysis, outcomes across the included studies were examined for evidence of publication bias using funnel plots. 

**Table1:** Initial features of trials

**Diarrhea** **duration**	**Concomitant treatment **	**Control group **	** Probiotic treatment**				**Age range **(Months)	**Location**	**Study**
			**Vehicle**	**Duration(day)**	**Dose (CFU)**	**Strain**			
< 3 d	ORS	Placebo (non-probiotic yogurt)	yogurt	3 times per day	10^7^	L. acidophilus &Bifidobacteria	6-72	Iran	Abbaskhaniyan et al. 2012 [1]
< 3 d	ORT	Placebo	Tablet dispersed in water	Twice a day for 5 days	6 × 10^7^	Lactobacillus Sporogenes (Bacillus coagulans)	6-24	India	Dutta et al. 2011 [20]
< 96 h	ORS	Oral and / or parenteral solutions.		Once a day for 5 days.	250 mg	Saccharomyces boulardii	1-28	Turkey	Dalgic et al. 2011 [3]
	ORT	Placebo	Dissolved in water	Twice a day for 5 days		Group A. Saccharomyces boulardii, Group B. A compound Containing L. acidophilus & L. rhamnosus& B. longum& S. boulardii	1-23	Bolivia	Grandy et al. 2010 [9]
< 7 days	ORS or intravenous solution with Ringer’s lactate	Placebo	Capsule contained 5 ml of sterile Nacl 0.9 %	3 times for 3 days	5 × 10^9^	Lactobacillus casei strain GG	4-24	Australian	Ritchie et al. 2010 [46]
≤ 3 d	Standard therapy (ORS)	placebo	Sachets	3 times for 14 days	1 sachet	Probiotic (Bifilac)	3-36	India	Narayanappa D, 2008 [47]
1 - 5 d		Placebo	Freezed dried	Twice daily for 5 days	1.2 × 10^10 ^	Lactobacillus rhamnosus GG	2 – 72 (Rotavirus infection : 45% )	Poland	Szymanski et al. 2006 [48]
< 48 h	ORS	Placebo (Whey-protein / skim-milk powder blend)	ORS	Twice daily for 5 days	5× 10^10 ^	L. paracasei	4-24	Bangladesh	Sarker et al. 2005 [49]
≤ 7 d		Placebo	Consisted of lyophilized	Twice daily for 5 days	4× 10^10^	L. rhamnosus 19070-2 &L.reuteri DSM 12246	6-36	Denmark	Rosenfeldt et al. 2002 [50]
	ORS	ORS + Placebo	ORS		At least 1010 CFU/250ml	Lactobacillus GG	1-36		Guandalini et al. 2000 [51]
< 5 days	ORT for the first 4 hours. Second, undiluted formula or breast milk fed with ORS.	Placebo	Sachet	Twice daily for 5 days	5× 10^9^	*L. acidophilus* LB	3-24	Thailand	Simakachorn et al. 2000 [52]
< 5 days	ORT	Placebo (the cellulose powder)	Bag of dried power in 5 ml of water & mixed with ORS or another drink or food	Twice daily for 5 days	5 × 10^9^	LGG	1-36	Russia	Shornikova et al. 1997 [53]
		Placebo		Once a day up to 5 days	Small dosage (10^7^CFU) Large dosage (10^10^CFU)	*L. reuteri*	6-36		Shornikova et al. 1997 [44]
<7days	ORT twice	Placebo (fermented-then-pasteurized yogurt, with <10^3^cfu lactic acid bacteria)	Fermented milk product	125 gr twice daily	10^10-11 ^	*L. casei* strain GG	7-37	Finland	Kaila et al. 1992 [39]

a CFU, colony-forming units

A priori Subgroup analysis was planned to distinguish the modification of reductions in diarrhea by LGG type in LGG probiotics group and non-LGG probiotics groups. The Stata 9 software (Stata Corp, College Station, Tex) was used for statistical analysis to perform the meta-analysis of the RCTs with random effect. Continuous outcomes (duration of diarrhea) are presented as standardized mean difference (SMD) between the probiotic treatment and controls with 95% confidence intervals. Heterogeniuity of data was tested by I^2 ^index and sources of heterogeneity were identified through accomplishing subgroup analysis.

## Results

The literature review resulted to 1224 documents, of which 14 were assessed for eligibility and were included in the meta-analysis. Finally, a total of 1149 patients were included in these studies. We categorized these trials as kind of probiotics to three subgroup analysis, l*actobacillus rhamnosus* GG and non-LGG, and all trials were categorized as the other group (n=14). Major strain of probiotic used was *L. rhamnosus* GG. The age range of patients were 1-72 months. In the major trials, they administered the probiotics available either as capsules, tablets, powders, or granules. In two trials they used them by premixing with a selection of food vehicle such as fermented milk or yogurt. 

**Figure 2:**
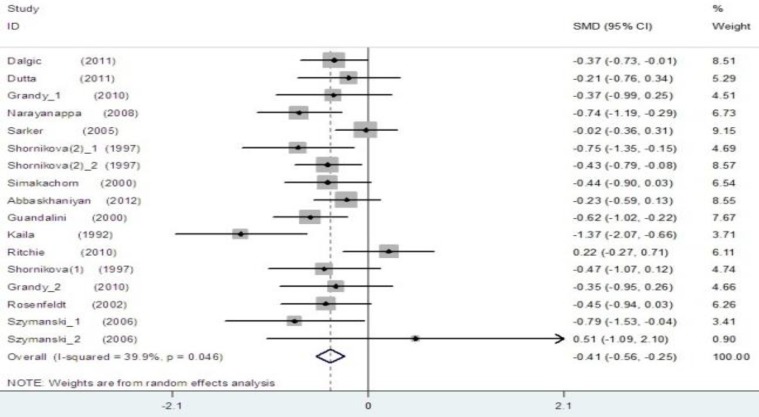
The effect size for the overall effects of probiotics in the duration of diarrhea

**Figure 3:**
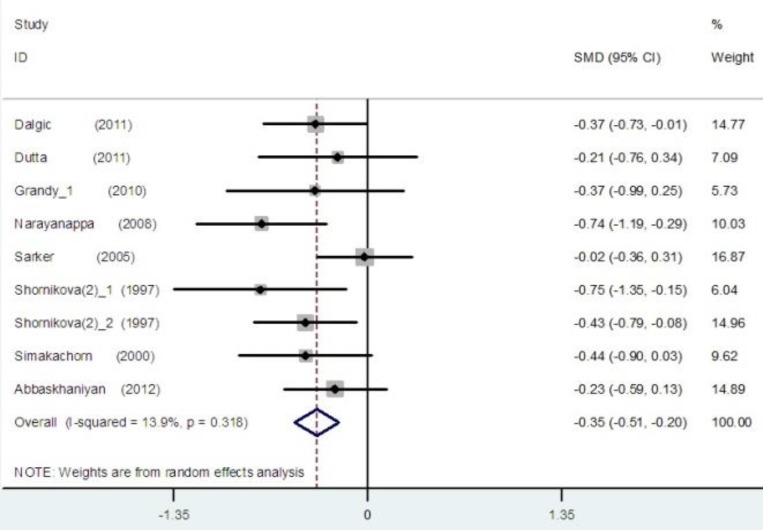
The effect size for effects of non-LGG probiotics in the duration of diarrhea

**Figure 4:**
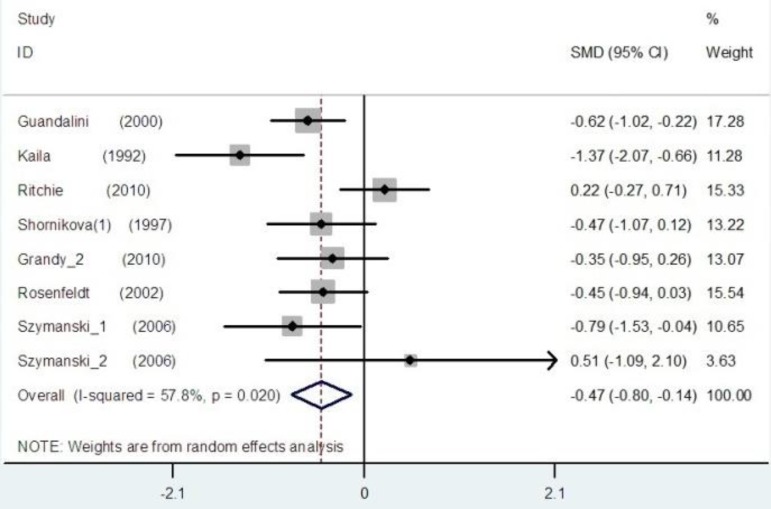
The effect size for effects of non-LGG probiotics in the duration of diarrhea

**Figure 5:**
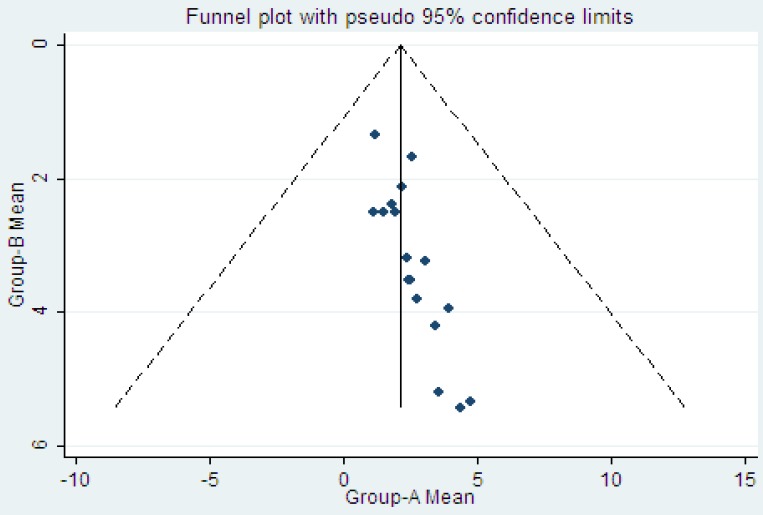
Funnel plot for enrolled studies

The pooled estimate of efficacy of probiotics in the prevention of disease yielded in all studies a mean difference of 0.41 (95% CI -0.56 to –0.25; p<0.001) and a heterogeneity (I2) of 39.9% (figure 2). The pooled estimate of efficacy of LGG probiotics and others had significant reduction in duration of diarrhea and non-LGG probiotics show low I2 score (figures 3 and 4). Among trials with the data on the effects of LGG, two results had positive point estimates and six results attained statistical significance with an overall reduction of 0.47 (95% CI -0.80 to -0.14; P=0.020) and a heterogeneity (I2) of 57.8%. The funnel plot for publication bias had an asymmetrical distribution (figure 5). Among trials, administering probiotics available as capsules, tablets, granules and powders with a selection of food vehicle had no significant difference in the protective point estimates. And the protective effect by mode of delivery was not influenced by the patient’s age.

## Discussion

In this meta-analysis, the efficacy of probiotics in the treatment of acute rotavirus diarrhea in children was investigated by reviewing several studies, which yielded contradictory results. The results of the present study showed that probiotics had a positive effect in reducing the duration of acute rotavirus diarrhea in children in comparison with control. Previously published meta-analyses used from studies that focused on researches related to high-income countries in the hospital, were restricted to infants and children (26, 41). The results of one-meta-analysis which compared lactobacillus rhamnosus 66 with placebo, demonstrated reduction of healthcare-associated diarrhea (42). We selected 14 trials according to inclusion criteria and surveyed the duration agent in these trials. The major trials had protective point estimates; most of them attained statistical significance. Three trials had statistically non-significance and non-protective point estimates. Significant differences in effectiveness have been observed in different species. This can be seen in several illustrations of these RCTs that Rosenfeldt et al. showed that *lactobacillus rhamnosus* and *lactobacillus reuteri* improved acute diarrhea in hospitalized children and reduced the duration of rotavirus expulsion (42). In line with the recent finding, Szajewska et al. noted that the use of probiotics can reduce the period of diarrhea, especially rotavirus diarrhea between 20 to 24 hours (25). In one such study reported that the b*ifidobacterium lactis* had a complementary role in the treatment of rotavirus gastroenteritis and other probiotics may also have a positive effect in rotavirus diarrhea compared with placebo (43). Moreover, the efficacy of *lactobacillus reuteri* in hospitalized children with rotavirus diarrhea was demonstrated in one study. These bacteria shortened the duration of diarrhea with a dose-dependent effect (44). Lactobacillus GG (3× 10^9^ cfu/g twice daily for a maximum of 6 d) reduced the first half period of diarrhea in outpatient children and significantly reduced rotavirus shedding (45). Another study indicated that there is a dose-response relevance involved. Although these differences were statistically significant, but further studies are still recommended.

In conclusion, the value of meta-analysis is that it provides an instrument to incorporate trials with the above differences and reach to a pooled estimate of the efficacy of different probiotics. The extracted data from the RCTs demonstrated adequate evidence for the positive significant effect of probiotics in the reduction of duration of acute rotavirus diarrhea. To prove this evidence requires such research with identical dosage and methodology to be performed before further conclusions can be drawn.
